# Perspectives on the basis of seizure-induced respiratory dysfunction

**DOI:** 10.3389/fncir.2022.1033756

**Published:** 2022-12-20

**Authors:** Daniel K. Mulkey, Brenda M. Milla

**Affiliations:** Department of Physiology and Neurobiology, University of Connecticut, Storrs, CT, United States

**Keywords:** cortical-brainstem connectivity, seizure propagation, spreading depolarization (SD), SUDEP (sudden unexpected death in epilepsy), apnea

## Abstract

Epilepsy is an umbrella term used to define a wide variety of seizure disorders and sudden unexpected death in epilepsy (SUDEP) is the leading cause of death in epilepsy. Although some SUDEP risk factors have been identified, it remains largely unpredictable, and underlying mechanisms remain poorly understood. Most seizures start in the cortex, but the high mortality rate associated with certain types of epilepsy indicates brainstem involvement. Therefore, to help understand SUDEP we discuss mechanisms by which seizure activity propagates to the brainstem. Specifically, we highlight clinical and pre-clinical evidence suggesting how seizure activation of: (i) descending inhibitory drive or (ii) spreading depolarization might contribute to brainstem dysfunction. Furthermore, since epilepsy is a highly heterogenous disorder, we also considered factors expected to favor or oppose mechanisms of seizure propagation. We also consider whether epilepsy-associated genetic variants directly impact brainstem function. Because respiratory failure is a leading cause of SUDEP, our discussion of brainstem dysfunction focuses on respiratory control.

## Introduction

**Figure 1:**
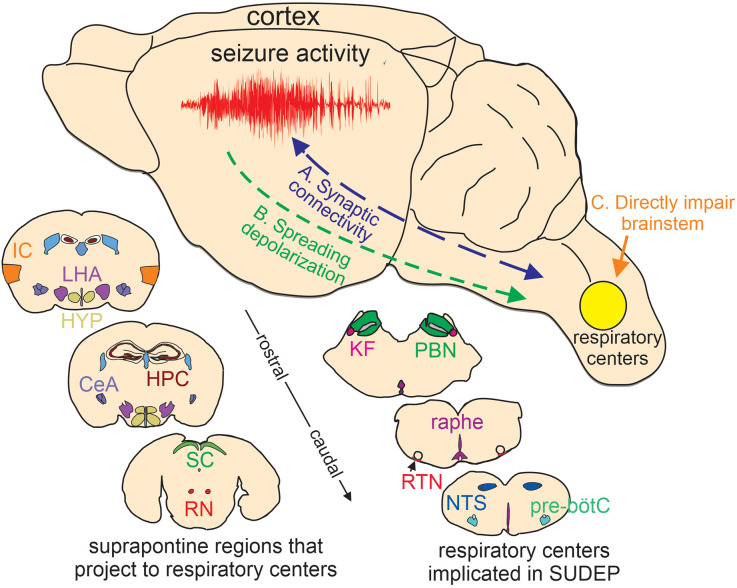
Putative basis of brainstem dysfunction in epilepsy. **(A)** Cortical-brainstem connectivity provides an anatomical basis for seizure propagation to the brainstem. Likewise, brainstem-cortical projections have been shown to synchronize cortical network activity and so may influence seizure propensity. **(B)** Spreading depolarization can propagate through contiguous tissue and if it reaches the brainstem will likely result in respiratory failure. **(C)** certain epilepsy-associated ion channel mutations are expressed in the brainstem where they directly impair respiratory function. Lower insets, cartoon coronal sections show the relative location of suprapontine regions that project to brainstem respiratory centers implicated in SUDEP (Harper et al., 1998; McKay et al., 2003; Rosin et al., 2006; Yang et al., 2020; Trevizan-Baú et al., 2021; Feinstein et al., 2022; Leitner et al., 2022). IC, insular cortex; LHA, lateral hypothalamus; HYP, hypothalamus; CeA, central amygdala nucleus; SC, superior colliculus; RN, red nucleus; KF, kölliker-fuse; PBN, parabrachial nuclei; raphe, medullary raphe nucleus; RTN, retrotrapezoid nucleus; NTS, nucleus of the solitary tract; pre-bötC, pre-bötzinger complex. Note, that the medullary raphe is part of the reticular activating system.

Epilepsy is a chronic disease associated with uncontrolled brain activity that results in recurrent seizures. Approximately 50 million people globally have epilepsy and people with this condition have a two-three-fold higher mortality rate than the general public. Sudden unexpected death in epilepsy (SUDEP)- defined as death in people with epilepsy that are not caused by injury, drowning, or other known reasons- is a leading cause of death in epilepsy patients (Pathak et al., 2022) and is second only to stroke in years of potential life lost to neurological disease, thus making SUDEP a significant public health problem (Thurman et al., 2014). Despite their potential lethality, most seizures are not fatal, and so a frequent question posed by family members of SUDEP victims is “what was it about that [final] seizure that resulted in death”^1^? Considering seizures typically originate in the cortex and lethality involves disruption of autonomic (Thijs et al., 2021) and respiratory (Teran et al., 2022) function at the level of the brainstem, to address this question, we discuss two likely mechanisms by which cortical seizure activity propagates to the brainstem. We also consider whether the expression of epilepsy-associated genes in the brainstem contributes to epilepsy-associated cardiorespiratory dysfunction (Figure 1). It is our contention that SUDEP is a heterogenous process involving different mechanisms depending on the underlying cause of seizure activity. Highlighted here are what we consider the most likely mechanisms by which cortical seizure activity might propagate to the brainstem; however, it is also important to recognize that other regions and polysynaptic pathways may contribute to descending seizure propagation.

## I. Descending Seizure Propagation Through Synaptic Connectivity

Essential components of the respiratory circuit are located in the brainstem and include inspiratory rhythmogenic neurons in the pre-bötzinger complex (pre-bötC; Smith et al., 1991), neurons in the retrotrapezoid nucleus (RTN; Mulkey et al., 2004) and medullary raphe (Richerson, 2004; Ray et al., 2011) that regulate breathing in response to changes in CO_2_/H^+^ (i.e., function as respiratory chemoreceptors), parabrachial neurons that modulate inspiratory-expiratory phase transitions and integrate chemoreceptor, visceral and arousal information between the forebrain and brainstem (Kaur and Saper, 2019), and respiratory motor neurons that serve as the final common output for the respiratory system (Fogarty et al., 2018). These respiratory centers also receive input from suprapontine regions including the insular cortex (McKay et al., 2003), hippocampus (Harper et al., 1998), and amygdala (Feinstein et al., 2022); in humans, stimulation of these regions results in cessation of breathing (Ochoa-Urrea et al., 2020), presumably to allow for voluntary and emotional control of ventilation (Bondarenko et al., 2014; Ashhad et al., 2022). Evidence also suggests communication between brainstem respiratory centers and suprapontine structures is bidirectional; ascending respiratory activity entrains cortical and limbic network oscillations that are thought to be important for emotion and memory consolidation (Herrero et al., 2018; Karalis and Sirota, 2022). Furthermore, brainstem projections from the reticular formation to the thalamus and cortex *via* the reticular activating system modulate sleep-wake transitions and arousal (Kovalzon, 2016). In the context of epilepsy, cortical-brainstem connections provide an anatomical substrate for cortical seizure propagation to the brainstem, and as such, have long been implicated in seizure-induced cardiorespiratory dysfunction (Frysinger and Harper, 1990).

The amygdala stands out as a hub of the so-called brainstem-homeostatic forebrain connectome (Edlow et al., 2016). This region is located in the temporal lobe and sends extensive inhibitory projections to brainstem respiratory centers where it is thought to regulate fear-related respiratory responses (Nardi et al., 2009; Feinstein et al., 2022), particularly to external perceived threats but not necessarily interoceptive threats (Feinstein et al., 2013; for review see Feinstein et al., 2022). The amygdala is also highly susceptible to seizure activity (Aroniadou-Anderjaska et al., 2008), and animal models (Totola et al., 2019), as well as clinical work from pediatric (Rhone et al., 2020) and adult (Dlouhy et al., 2015; Lacuey et al., 2017; Nobis et al., 2018) epilepsy patients, showed that stimulation or seizure activation could elicit apnea. Consistent with its lack of involvement in interoceptive threats (Feinstein et al., 2022), amygdala-evoked apneas did not occur in conjunction with dyspnea (Dlouhy et al., 2015). Curiously, amygdala-evoked apneas are dependent on attention and nasal breathing (Nobis et al., 2018). This is interesting because cortical respiratory rhythms also depend on nasal breathing (Zelano et al., 2016), suggesting there is a hierarchical organization to cortical-brainstem communication where conscious effort through attention or elicited by mouth breathing can override coordinated activity between regions. This also suggests interventions that facilitate mouth breathing might limit seizure-induced apnea.

It should be noted that the amygdala is composed of multiple sub-nuclei and only a subset of which contribute to the respiratory activity. For example, stimulation of the basolateral, basomedial, and central regions consistently resulted in apnea, whereas stimulation of the more lateral amygdala failed to affect breathing (Rhone et al., 2020). Therefore, not all amygdala seizures result in apnea (Park et al., 2020). It is also worth mentioning that amygdala stimulation elicited apnea even during sleep (Nobis et al., 2018) when SUDEP occurs most frequently (Nobili et al., 2011). By contrast, another putative SUDEP mechanism, namely spreading depolarization (SD), is less likely to be favored during sleep (see next section below). In any case, these mechanisms are not mutually exclusive, but rather may occur simultaneously and in a synergistic manner. For example, SD will result in high extracellular potassium ([K^+^]o) and this has been shown to facilitate excitatory more so than inhibitory synaptic transmission (Rasmussen et al., 2020), thus promoting synaptic seizure propagation. Likewise, excitatory synaptic transmission has been shown to facilitate SD in a seizure-related mouse model of familial hemiplegic migraine type-1 (Tottene et al., 2009). Furthermore, SUDEP-prone mouse lines showed cortical epileptic activity that correlated with abnormal brainstem electroencephalographic (EEG) oscillations and suppression of brainstem activity (Gu et al., 2022), suggesting cortical-brainstem connectivity facilitated SD propagation to the brainstem.

In addition to individual ictal events causing cardiorespiratory arrest as described above, it is also possible that repeated cortical seizures cause maladaptive changes to the forebrain and brainstem respiratory circuity that render the respiratory system vulnerable to failure. In this case, epilepsy patients are expected to exhibit background cardiorespiratory abnormalities. Indeed, interictal cardiorespiratory problems are common in epilepsy (Barot and Nei, 2019). Typical background autonomic and respiratory symptoms exhibited by Dravet syndrome patients and partly recapitulated in animal models of this disease include diminished heart rate variability, bradycardia, and hypoventilation with increased apnea (Delogu et al., 2011; Kim et al., 2018). The possibility the brainstem is disrupted in epilepsy is also supported by anatomical evidence showing patients with focal epilepsy have diminished brainstem volume (Mueller et al., 2018) including loss of both neurons (Patodia et al., 2021) and astrocytes (Patodia et al., 2019) in respiratory control centers. Evidence also suggests that seizure activity can alter synaptic connectivity between cortical and brainstem structures, and such circuit level changes may alter brainstem function and perpetuate excitotoxicity-related damage (Armada-Moreira et al., 2020). For example, amygdala neurons located ipsilateral to the temporal lobe seizure foci exhibited a stronger convergence of cardiorespiratory information compared to neurons on the contralateral side (Frysinger and Harper, 1990), thus indicating seizure activity enhanced excitatory coupling between cortical and brainstem respiratory areas. Nevertheless, the possibility that altered cortex-brainstem connectivity contributes to brainstem dysfunction or cell death remains unknown. From a bottom-up perspective, it is conceivable that enhanced respiratory-driven oscillations in cortical regions might increase neural network synchronization and seizure propensity.

It has also been speculated that seizure-induced amygdala-dependent apnea is independent of the underlying cause or type of epilepsy (Rhone et al., 2020). However, considering the central nucleus of the amygdala provides a primary output of this region to the brainstem (Feinstein et al., 2022) and since neurons in this region are mostly inhibitory and suppress breathing presumably by synaptic inhibition, it seems unlikely such a mechanism would be effective in forms of epilepsy associated with loss of inhibitory tone. Consistent with this, amygdala neurons that make monosynaptic projections to the parabrachial pneumotaxic center are hypoexcitable in a mouse model of Dravet syndrome (Yan et al., 2021). Also, inhibitory neurons in the amygdala appear prone to seizure-induced damage (Tuunanen et al., 1996), which conceivably will favor epileptogenesis in the amygdala and nearby hippocampus but is less likely to increase inhibitory bombardment of brainstem respiratory centers.

**Figure 2:**
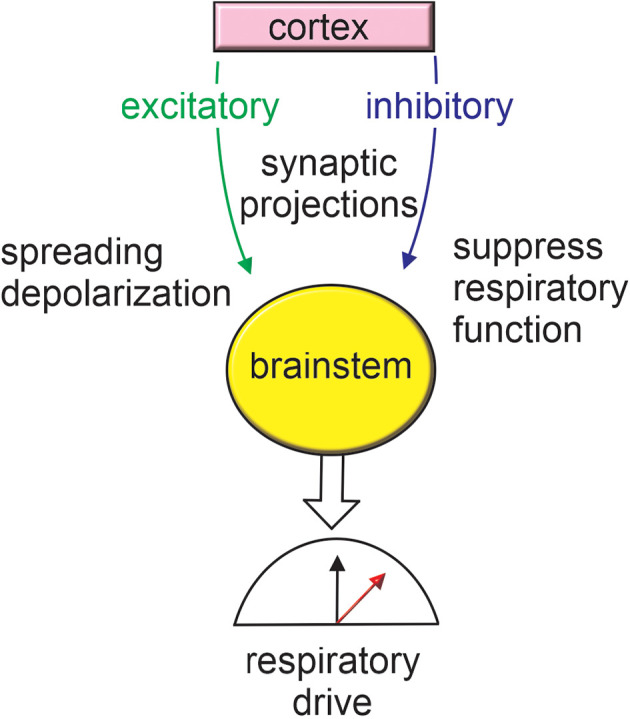
Cortex-brainstem connections provide a framework for seizure-induced respiratory failure. Seizure activation of inhibitory cortical projections to brainstem respiratory centers has been shown to result in respiratory arrest. Seizure activity may also stimulate excitatory brainstem projections thereby resulting in metabolic stress and favoring the propagation of spreading depolarization. For more information on anatomical regions refer to Figure 1.

In sum, seizure activation of cortical to brainstem inhibitory projections can disrupt cardiorespiratory function and contribute to SUDEP (Figure 2). Repeated activation of cortical-brainstem circuits may also alter network connectivity in maladaptive ways that compromise cardiorespiratory control, favor seizure propagation to the brainstem or increase synchronized cortical activity to increase seizure propensity.

## Ii. Spreading Depolarization

SD is a pathological event associated with migraine headache, ischemic, or traumatic brain injury and epilepsy (for reviews see Pietrobon and Moskowitz, 2014; Cozzolino et al., 2018). It is thought to be triggered by a severe depolarization that leads to large-scale loss of ion and transmitter homeostasis. In particular, a pronounced increase in [K^+^]o and glutamate can initiate a self-propagating wave of depolarization and cytotoxic edema (Hinzman et al., 2016; Hubel et al., 2017). The ability of such a wave to propagate into and through contiguous tissue is strongly influenced by ongoing neural activity and metabolic status (Aiba and Noebels, 2015). SD is followed shortly thereafter by a wave of neural inactivation (presumably caused by depolarizing block) that results in depression of EEG activity as frequently observed following generalized tonic-clonic seizures (GTCS; Surges et al., 2011). In the cortex, this so-called spreading depression may serve a protective role by limiting further seizure activity (Tamim et al., 2021). However, if such an event were to occur in the brainstem it is expected to have a negative impact on cardiorespiratory control. Consistent with this, GTCS are the most common type of seizure associated with SUDEP (Ryvlin et al., 2019), and mechanisms underlying GTCS are thought to involve dysregulation of the ascending reticular activating system and descending reticulospinal projections to result in characteristic features of GTCS including loss of consciousness and muscle convulsions (Sedigh-Sarvestani et al., 2015).

In epilepsy patients it is unclear whether post-ictal EEG suppression is an independent risk factor SUDEP (Ryvlin et al., 2019); however, pre-clinical experiments using monogenic SUDEP models showed that brainstem SD correlated with cardiorespiratory failure and mortality. For example, cortical-evoked seizures in two SUDEP mouse models (Kv1.1 null and *Scn1a*^R1407X/+^ loss of function) resulted in brainstem SD and cardiorespiratory arrest (Aiba and Noebels, 2015). Similar results were also observed in mice expressing a Ca_v_2.1 gain of function mutation associated with familial hemiplegic migraine type 1 (*Cacna1a*^S218L/+^; Jansen et al., 2019) and mice expressing a ryanodine receptor-2 gain of function mutation associated with catecholaminergic polymorphic ventricular tachycardia (Aiba et al., 2016). Also, in *Cacna1a*^S218L/+^ mice the superior colliculus, a midbrain structure that receives input from both the cortex and brainstem, was particularly effective at propagating seizure-induced SD to the brainstem (Cain et al., 2022), suggesting anatomical connectivity may facilitate SD.

Interestingly, expression of certain epilepsy-associated channel variants in the cortex but not the brainstem can cause cortical seizures and SD but in the absence of increased mortality. For example, Kcnq2 channels produce a subthreshold K^+^ conductance (Abbott, 2020) and loss of *Kcnq2* function is associated with neonatal epileptic encephalopathy (Orhan et al., 2014; Kim H. J. et al., 2021). Conditional deletion of *Kcnq2* from forebrain excitatory neurons (*Emx1*^Cre/+^::*Kcnq2*^f/f^; *Kcnq2* cKO) resulted in cortical seizures and SD. Despite this, only a small subset of these animals died prematurely (Aiba and Noebels, 2021). However, a caveat to these experiments is that *Emx1*^cre/+^ is not restricted to cortical excitatory neurons but rather is also expressed by peripheral autonomic ganglia that provide modulatory feedback to cardiorespiratory centers (Ning et al., 2022). Excluding potential confounding effects of peripheral nerves, these results suggest compromised brainstem function is required for cortical seizure- or SD-induced respiratory arrest and premature death (see Section “III. Direct effect of epilepsy associated mutations on brainstem function” below for more detail).

The initiation phase of SD is dependent on the concentration of ions and transmitters in the extracellular space which are themselves inversely related to extracellular volume (ECV). Also, ECV increases during sleep (Ding et al., 2016), therefore, we speculate the threshold for SD induction will be higher during sleep when SUDEP is thought to occur most frequently (Nobili et al., 2011). Consistent with this, spontaneous cortical SD in *Kcnq2* cKO mice (Aiba and Noebels, 2021), as well as seizure-induced death in Kv1.1 null mice (Moore et al., 2014) and *Scn1a*^R1407X/+^ mice (Teran et al., 2019), occurred primarily during the dark/active state when ECV is expected to be minimal and the impact of [K^+^]o on neural activity is most favored (Ding et al., 2016). Therefore, these results are consistent with the involvement of high [K^+^]o as a key determinant of SD threshold and propagation. However, these results are not consistent with clinical evidence suggesting SUDEP occurs primarily during sleep (Buchanan et al., 2021). Note that once SD has been initiated the corresponding cellular edema is expected to negate this issue; thus, SD propagation is not expected to be sleep-wake state dependent.

Seizure events can deplete energy availability, thus limiting ion and transmitter buffer capacity and lowering SD induction threshold (Major et al., 2020). Consistent with this, Kv1.1 null and *Scn1a*^R1407X/+^ models showed a low threshold for SD elicited by metabolic stress. Furthermore, repeated seizures may facilitate pathological remodeling that can lead to progressively more severe outcomes. For example, mice that express the *Scn1a* loss of function variant R1648H exhibit a mild/asymptomatic phenotype under resting conditions that can be transformed into a severe phenotype by subjecting the mice to heat- or chemoconvulsant-induced seizures (Dutton et al., 2017; Salgueiro-Pereira et al., 2019). This study also showed that wild type mice subjected to the same seizure induction protocol did not develop a severe seizure phenotype. These results suggest loss of *Scn1a* function lowered the seizure threshold, as expected, and is required for remodeling following repeated seizures that can lead to severe phenotypes and SUDEP. However, contrary to this, early work with chemoconvulsant models of epilepsy suggests frequent seizures can confer resistance to SD. For example, a pentylenetetrazol rat model of epilepsy showed that frequent seizures increased the SD threshold (Koroleva et al., 1993). Furthermore, patients with chronic epilepsy and pilocarpine-treated rats exhibited a similar high [K^+^]o threshold for SD (Maslarova et al., 2011). Based on this, it was speculated that chronic seizures promote a compensatory increase in [K^+^]o buffering capacity.

A critical function of astrocytes is to regulate extracellular ion and transmitter homeostasis and as such are important determinants of [K^+^]o buffering. The dynamics of [K^+^]o are complex and depend on several factors. Here, we focus on inward rectifying Kir4.1 channels because these are the main determinant of astrocyte resting membrane potential (by K^+^ efflux) and can serve as a conduit for K^+^ uptake when the reversal potential for K^+^ is depolarized to resting membrane potential as can occur during increased neural activity. Glutamate uptake by astrocytes is also an electrogenic process favored at more negative membrane potentials (Grewer and Rauen, 2005). Astrocytes are also highly sensitive to seizure activity and in chronic epilepsy these cells are known to proliferate (gliosis) and transition into a pro-inflammatory state (so-called reactive astrocyte) characterized by the release of cytokines and growth factors that can increase seizure propensity or promote tissue repair (for review see Wetherington et al., 2008; Verhoog et al., 2020). Although there is some evidence suggesting Kir4.1 expression increased in a pilocarpine model of temporal lobe epilepsy (Nagao et al., 2013) and a mouse model of Dravet syndrome (Miljanovic et al., 2021), most studies suggest the opposite, that astrocyte Kir4.1 channel expression is diminished in epilepsy (Kinboshi et al., 2020; Ohno et al., 2021). Indeed, loss of function variations (missense and nonsense mutations) in *KCNJ10* (the gene encoding Kir4.1) causes an epileptic disorder known as EAST/SeSAME syndrome (Bockenhauer et al., 2009) characterized by early onset tonic-clonic seizures, sensorineural deafness, ataxia, intellectual disability, and electrolyte imbalance. Also, astrocyte Kir4.1 expression or function has been shown to be reduced in humans (Heuser et al., 2012; Steinhauser et al., 2012; Kitaura et al., 2018) and various animal models of epilepsy (Harada et al., 2013) including DBA/2 model of audiogenic seizures (Inyushin et al., 2010). Note that Kir4.1 channels also contribute to K^+^ buffering by oligodendrocytes, and loss of oligodendrocyte Kir4.1 channels also increases seizure activity (Larson et al., 2018). Also, loss of serotonergic signaling by raphe neurons contributed to seizure propensity and respiratory arrest in DBA/2 mice (Cervo et al., 2005). This is interesting because Kir4.1 channels can heteromerize with Kir5.1 to form a CO_2_/H^+^ sensitive conductance (Xu et al., 2000), and recent work showed that both Kir4.1 and Kir5.1 transcript are expressed by medullary serotonergic raphe neurons and so may contribute to CO_2_/H^+^ detection by these putative chemoreceptors (Puissant et al., 2017). Moreover, loss of Kir5.1 (*Kcnj16* gene) resulted in an audiogenic seizure phenotype with increased mortality in a rat model of salt-sensitive hypertension and chronic kidney disease (Manis et al., 2021), probably by a mechanism involving disruption of heteromeric Kir4.1/5.1 channels since Kir5.1 does not form functional homomeric channels (Pessia et al., 1996). Therefore, disruption of homo or heteromeric Kir4.1 channels may be a common substrate for breathing problems and seizure propensity.

As expected, astrocyte-specific deletion of *Kcnj10* also disrupted K^+^ and glutamate uptake and resulted in increased seizure activity and premature death (Djukic et al., 2007). Kir4.1 channels also colocalize with aquaporin-4 (Aqp4) water channels (Nagelhus et al., 2004) and Aqp4 knockout mice exhibited slowed [K^+^]o clearance (Amiry-Moghaddam et al., 2003) and longer duration seizures following neural stimulation (Binder et al., 2006). Therefore, disruption of Kir4.1 may impact Aqp4 function and consequently regulation of cell size and ECV. This may be important because the ability of astrocytes to influence neural activity by paracrine signaling or regulation of extracellular ions and transmitters is proximity-dependent and inversely related to ECV (Murphy et al., 2017).

It is also possible that the loss of Kir4.1 containing channels will facilitate the release of various neuroactive signaling molecules from astrocytes. In particular, inhibition of Kir4.1 chanenls in cultured astrocytes increased expression of brain-derived neurotrophic factor (BDNF; Kinboshi et al., 2017), a growth factor that signals through TrkB receptors to regulate neural growth, differentiation, and synaptic plasticity (Cowley et al., 1994; Meakin et al., 1999; Huang and Reichardt, 2003). This is of interest because BDNF is a potent modulator of epileptogenesis; BDNF expression reportedly increased in the brains of epileptic patients and animal models of epilepsy (Jankowsky and Patterson, 2001), whereas disruption of BDNF/TrkB signaling suppressed seizure activity in epileptic mouse models (Kokaia et al., 1995; Hagihara et al., 2005; Liu et al., 2013). Although the link between the loss of Kir4.1 and increased BDNF expression remains murky, pharmacological evidence implicates activation of the MAPK/ERK pathway (Kinboshi et al., 2017), possibly in response to the depolarization-induced increase in intracellular Ca^2+^. Together, these results suggest seizure-induced changes in astrocyte Kir4.1 expression is maladaptive and likely to contribute to epileptogenesis.

It is also important to recognize that increased Kir4.1 expression will not necessarily diminish seizure propensity. For example, *KCNJ10* gain of function variants that result in increased channel expression (p.R18Q), diminish proton-dependent inhibition (p.R348H) or increased channel conductance (p.V84M) are also associated with seizure-like behavior (Sicca et al., 2011, 2016). Mechanistically, it is hard to imagine how increased Kir4.1 channel function in astrocytes might promote seizures. One possibility is that increased Kir4.1 channel activity may increase [K^+^]o buffering kinetics, thereby limiting [K^+^]o build-up during increased activity. This mechanism may minimize depolarization-induced Na^+^ channel inactivation and allow neurons to fire at higher frequencies for longer periods of time (Niday and Tzingounis, 2018). Note that increased Kir4.1 expression is not expected to substantially decrease [K^+^]o because a prerequisite for K^+^ uptake by astrocytes is high [K^+^]o and a depolarized K^+^ reversal potential relative to resting membrane potential. As such, decreasing [K^+^]o will favor K^+^ efflux.

Another interesting mechanism by which increased Kir4.1 might favor seizure activity involves dysregulation of brain pH. Astrocytes express high levels of the electrogenic sodium bicarbonate cotransporter (NBC; Turovsky et al., 2016). The most common NBC isoform expressed by astrocytes has 1 Na^+^: 2 ${\text{HCO}}_{3}^{-}$ stoichiometry and a predicted reversal potential of around −100 mV (Mulkey and Wenker, 2011). This value is negative to astrocyte resting membrane potential, thus under normal conditions ${\text{HCO}}_{3}^{-}$ flux through the NBC is directed inward (Mulkey and Wenker, 2011). If this is the case, then increased expression of Kir4.1 is expected to hyperpolarize astrocyte membrane potential and decrease electrogenic ${\text{HCO}}_{3}^{-}$ transport, thereby resulting in extracellular alkalosis. This is significant because just 0.2 pH unit increase in extracellular pH can cause seizures (Schuchmann et al., 2006).

In sum, there is no doubt that SD, once initiated, can have profound effects on neural activity, and preclinical studies clearly implicate SD as a cause of seizure-induced mortality. However, the correlation between postictal generalized EEG suppression (PGES), which presumably also reflects SD, and SUDEP is a matter of debate in the literature. Some studies suggest there is a correlation between PGES and SUDEP (Lhatoo et al., 2010; Moseley and DeGiorgio, 2015), whereas other studies found PGES duration is not a risk factor for SUDEP (Surges et al., 2011; Lamberts et al., 2013). Also, sleep-wake changes in ECV are not expected to favor the initiation of SD during sleep when SUDEP usually occurs. Furthermore, although chronic seizure activity may result in compensatory cellular responses to limit SD, such adaptations are not likely to involve increased astrocyte Kir4.1 expression since most evidence indicates loss of this channel in epilepsy. For this same reason, astrocyte Kir4.1 channels may have some therapeutic potential in treating epilepsy, possibly by limiting SD. Consistent with this, certain antiepileptic drugs have been shown to stimulate Kir4.1 expression (Mukai et al., 2018).

## Iii. Direct Effect of Epilepsy Associated Mutations on Brainstem Function

**Figure 3:**
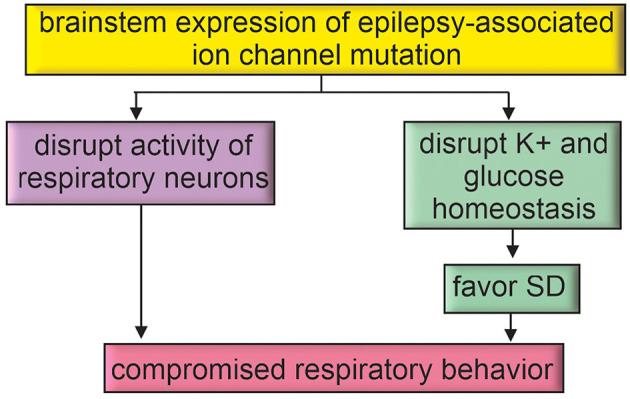
Epilepsy-associated genes may directly disrupt brainstem function. Brainstem expression of epilepsy-associated ion channel mutations may directly impact respiratory function or favor mechanisms for SD propagation to the brainstem.

In addition to promoting seizure activity in the cortex, epilepsy-associated genetic mutations may also be expressed in the brainstem (Kuo et al., 2019) where they increase SUDEP risk in seizure-dependent and -independent manners (Figure 3). For example, as noted above *Emx1^cre/+^::Kcnq2^f/f^* mice (*Kcnq2* cKO) showed spontaneous cortical seizures with SD but they did not die prematurely (Aiba and Noebels, 2021), suggesting the brainstem was protected from SD infiltration. Another study used a similar approach (*Emx1*^Cre/+^) to express a dominant negative *Kcnq2* variant (M547V) in forebrain pyramidal neurons (but with some off-target expression in astrocytes) of *Kcnq2* heterozygous knockout mice (C57BL/6 background); unlike *Kcnq2* cKO animals, these mice showed a severe phenotype including seizures and premature death (Kim E. C. et al., 2021). In this case, global *Kcnq2* haploinsufficiency appears sufficient to allow cortical seizures to disrupt brainstem function and result in mortality.

The possibility that brainstem expression of epilepsy-associated mutations increases the risk of mortality is supported by evidence that SUDEP can occur without overt seizure activity (Lhatoo et al., 2016). Pre-clinical animal experiments also support this possibility. For example, polymorphisms associated with DBA/2 mice (a common model of audiogenic seizures; De Sarro et al., 2017) appear to disrupt brainstem serotonergic signaling and contribute to seizure-induced cardiorespiratory failure. Specifically, DBA/2 mice express a single amino acid substitution in the gene encoding tryptophan hydroxylase-2 that results in limited serotonin production (Cervo et al., 2005), and this likely contributes to seizure-induced respiratory arrest since the systemic application of serotonin reuptake inhibitors improved seizure activity and related apneic events in DBA/2 mice (Faingold et al., 2014). DBA/2 mice also express a *Kcnj10* loss of function mutation that has been shown to disrupt Kir4.1-dependent maintenance of extracellular K^+^ and glutamate (Ferraro et al., 2004; Inyushin et al., 2010) and thus lower seizure threshold (Figure 2). It is also worth noting that Kir4.1 channels together with Kir5.1 may contribute to CO_2_/H^+^ chemosensation by serotonergic neurons (Puissant et al., 2017), thus loss of Kir4.1 could further compromise raphe chemoreception and worsen seizure-induced respiratory problems.

The retrotrapezoid nucleus (RTN) is another important respiratory chemoreceptor region implicated in SUDEP (Patodia et al., 2018). For example, in the context of Dravet syndrome (caused by loss of function mutations in *SCN1A*), we showed that *Scn1a* transcript is expressed by inhibitory parafacial neurons in the region of the RTN (Kuo et al., 2019). We also showed that inhibitory somatostatin (SST)-expressing neurons in the region of the RTN are inhibited by CO_2_/H^+^ and contribute to RTN chemoreception by disinhibition of CO_2_/H^+^-activated glutamatergic neurons (i.e., RTN chemoreceptors; Cleary et al., 2021). Therefore, in addition to causing cortical seizure activity, Dravet syndrome-associated *Scn1a* mutations may disrupt the inhibitory modulation of RTN chemoreception. Consistent with this, inhibitory neurons in the region of the RTN in slices from mice that express a loss of function *Scn1a* mutation (A1783V) conditionally in inhibitory neurons under the vesicular GABA transporter promoter (*Slc*32*a*^1*cre/*+^::*Scn*1*a*^*A*1783*V fl/*+^) showed lower basal activity compared to control cells and fired fewer action potentials in response to depolarizing current steps (Kuo et al., 2019). Consistent with a disinhibitory mechanism, chemosensitive RTN neurons in slices from *Slc*32*a*^1*cre/*+^::*Scn*1*a*^*A*1783*V fl/*+^ mice showed increased baseline activity and enhanced output in response to increases in CO_2_ (Kuo et al., 2019). However, at the whole animal level, Vgat^A1783V/+^ mice showed reduced respiratory activity in room air and a blunted ventilatory response to CO_2_ (Kuo et al., 2019). This outcome is not entirely unexpected because inhibitory signaling in the RTN (Cregg et al., 2017) and at other levels of the respiratory circuit (Baertsch et al., 2018) can facilitate respiratory output. Also, *Slc*32*a*^1*cre/*+^::*Scn*1*a*^A1783*V fl/*+^ mice have spontaneous seizures which, for reasons mentioned above, may propagate to the brainstem and disrupt respiratory control in a seizure-dependent manner. This later possibility is an important consideration since deletion of *Scn1a* only from forebrain inhibitory neurons also resulted in seizures and premature death (Cheah et al., 2012), suggesting in this mouse model that cortical seizure activity can cause brainstem dysfunction and SUDEP. This contrasts with evidence from *Kcnq2* cKO that as noted above exhibits cortical seizure activity that did not correlate with premature death (Aiba and Noebels, 2021). Both mouse models are maintained on a similar C56BL/6 background so the reason(s) for these divergent results remains unclear. That said, it is worth mentioning that disruption of *Scn1a* globally or conditionally only in forebrain inhibitory neurons caused sleep fragmentation with less non-rapid eye movement (NREM) sleep and more frequent waking episodes (Kalume et al., 2015). Although the relationship between sleep, sleep problems, and epilepsy have long been appreciated (Diaz-Negrillo, 2013; Wang et al., 2018), the basis for these associations is not clear. Based on evidence that regulation of the ECV is coupled to sleep-wake status (Ding et al., 2016) and decreased ECV positively correlates with neural activity (Walch et al., 2022), we speculate that disruption of sleep (as seen in Dravet syndrome; Kalume et al., 2015) will decrease ECV, lower seizure threshold and favor propagation of SD (Figure 2).

In sum, epilepsy-associated genes may be expressed by neurons or astrocytes in brainstem respiratory centers and so may contribute to background breathing problems that render the system vulnerable to failure. Altered neural activity or compromised astrocyte regulation of the extracellular milieu may also favor the propagation of SD into the brainstem.

## Author Contributions

DM drafted manuscript, drafted figures, and approved the final version. BM edited manuscript and figures, and approved the final version. All authors contributed to the article and approved the submitted version.

## Footnotes

^1^Partners Against Mortality in Epilepsy (PAME) hosts an annual meeting that brings together health care providers, basic scientists and families touched by epilepsy with the goal of improving our understanding of SUDEP. During previous PAME meetings we had the opportunity to talk with family members that lost a loved one to SUDEP. These interactions made a lasting impact on our perception of this disease and continue to motivate our work in this field.
